# Supplementary figures and images for: Changes and sex- and age-related differences in the expression of drug metabolizing enzymes in a KRAS-mutant mouse model of lung cancer
Source: PeerJ. 2020 Nov 18;8:e10182. doi: 10.7717/peerj.10182 (PMC7680056; doi:10.7717/peerj.10182)

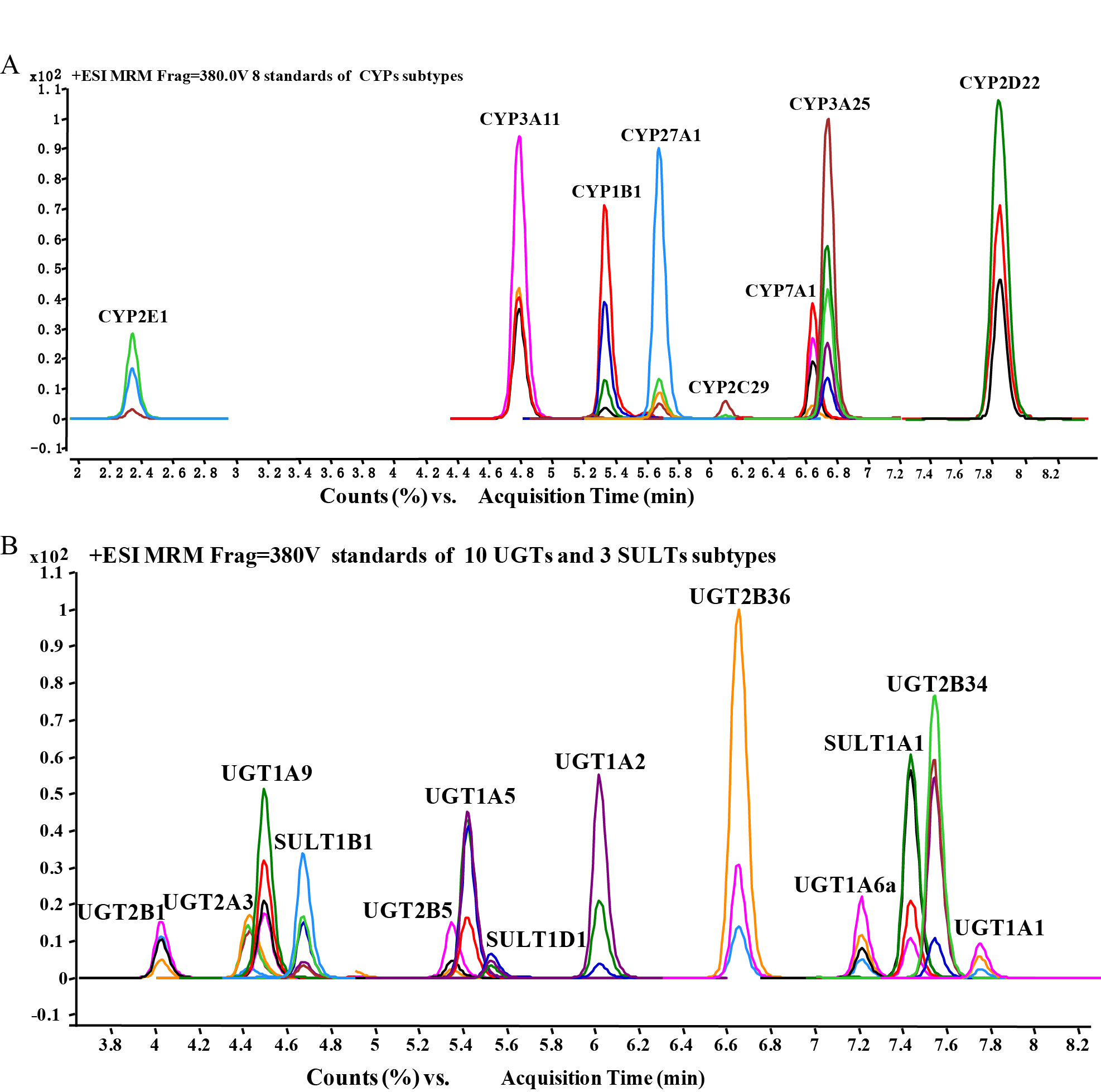

Supplement: Supplemental Information 1 — (A) the standards of CYPs. (B) the standards of UGTs and SULTs. [file peerj-08-10182-s001.png]

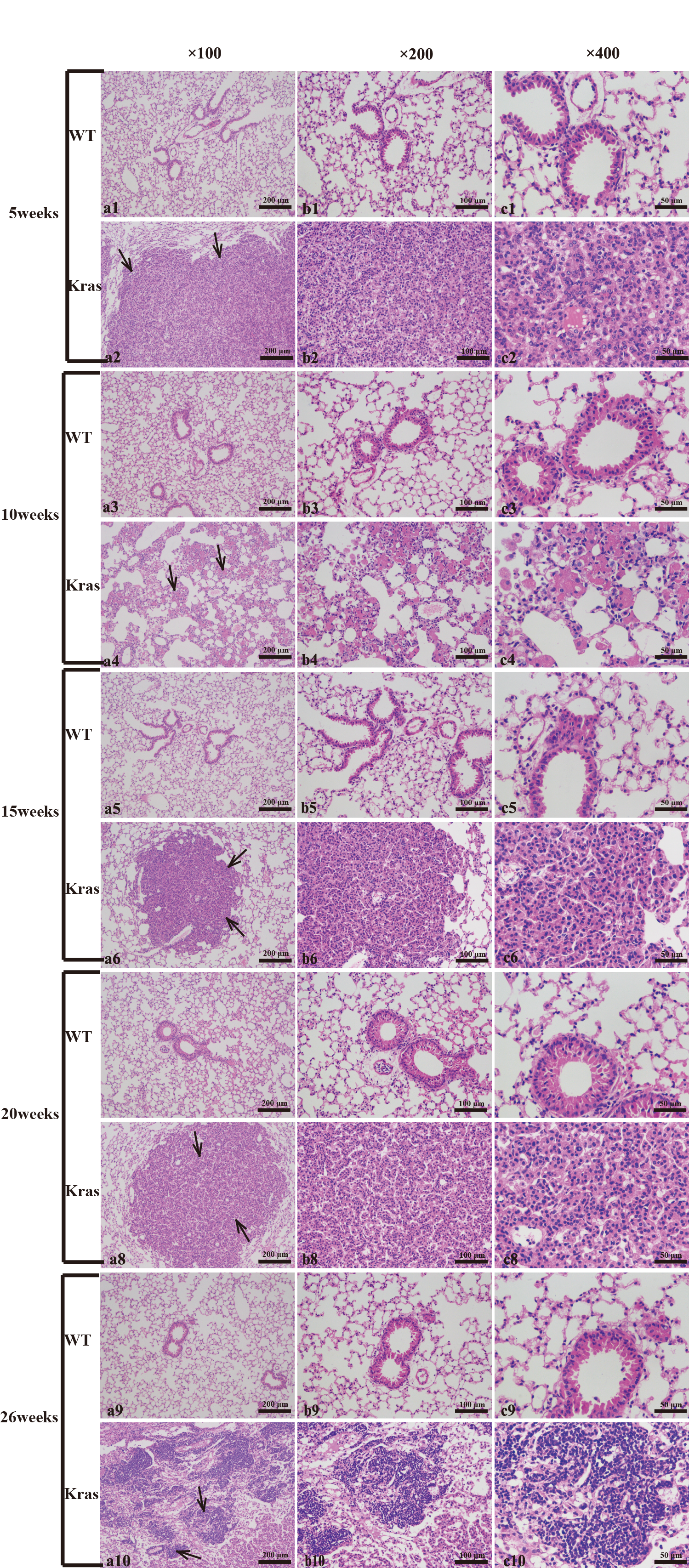

Supplement: Supplemental Information 2 — The arrows partly pointed out the hyperproliferative lung cells in KRAS mice. [file peerj-08-10182-s002.png]

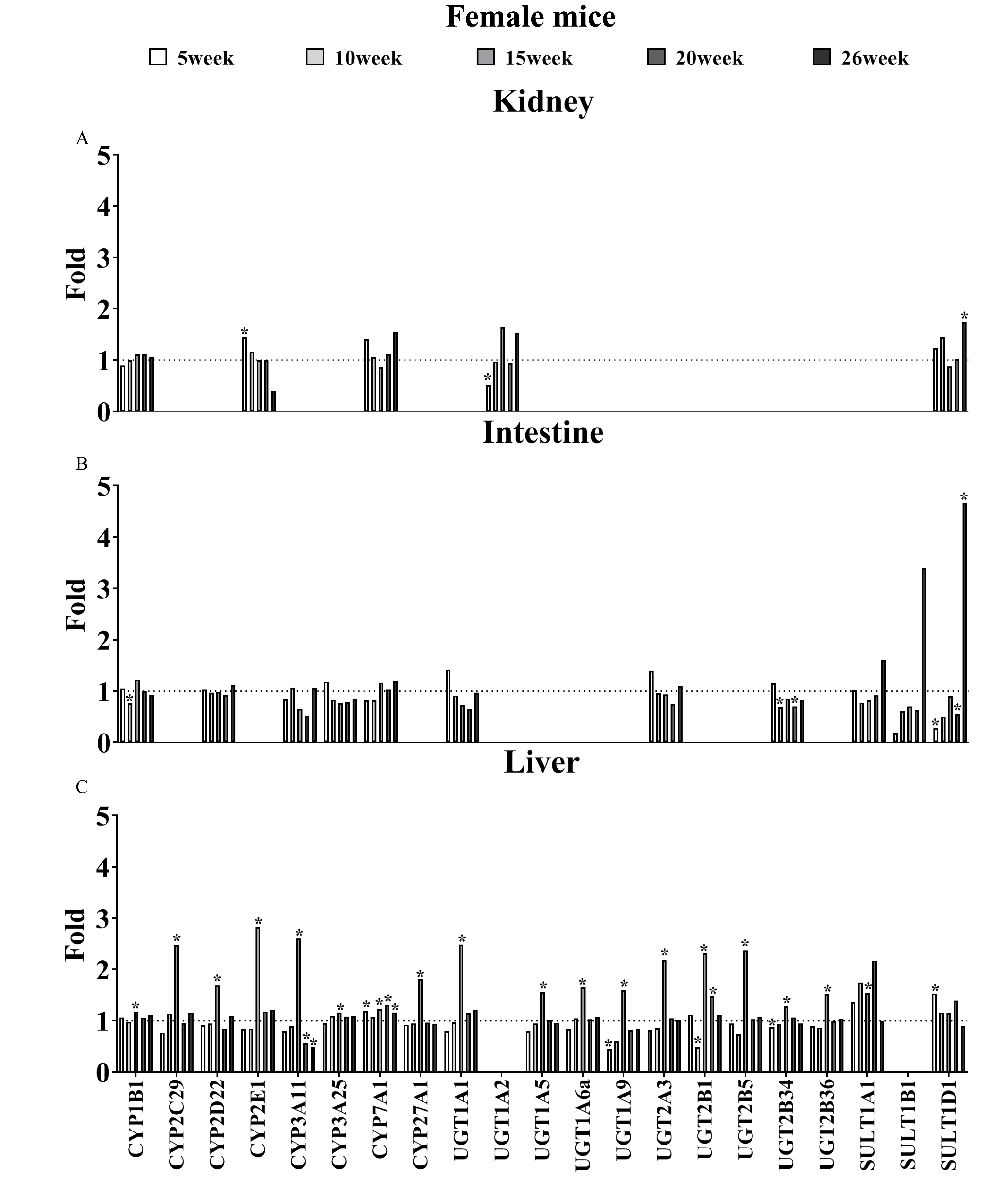

Supplement: Supplemental Information 3 — Protein levels in the female WT mice ( n = 5 ) were normalized to those in the female KRAS mice ( n = 5 ). The data were analyzed by independent sample t tests (for normally distributed data) and Mann-Whitney U analysis (for non-normally distributed data). The symbol “*” indicates a displayed significant difference between the male WT and KRAS mice at the same age, p < 0.05. [file peerj-08-10182-s003.png]

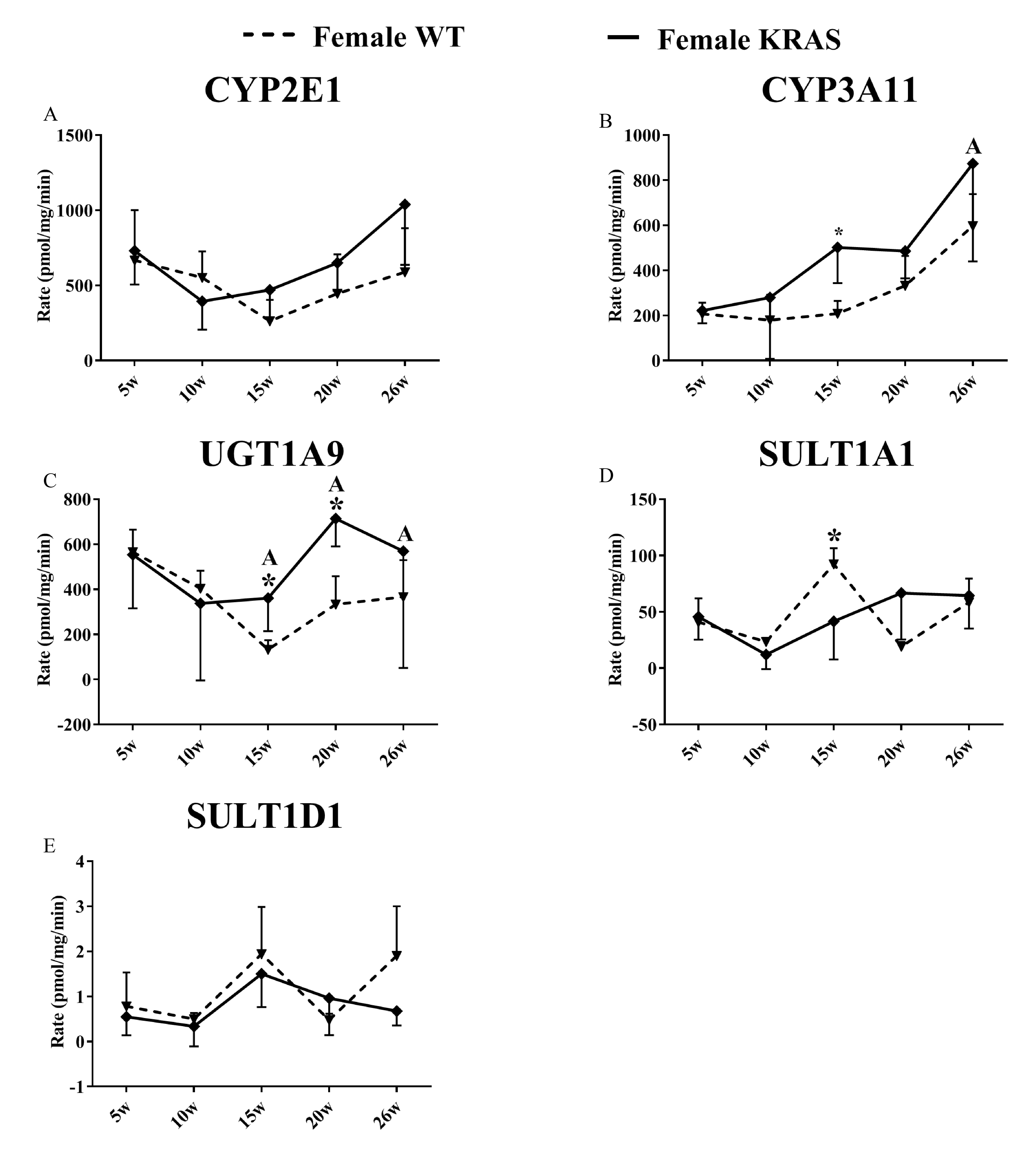

Supplement: Supplemental Information 4 — Each data point is presented as the mean ±SD. For the compare between KRAS and WT at the same age, the data were analyzed by independent sample t tests (for normally distributed data) and Mann–Whitney U analysis (for non-normally distributed data). The symbol “*” indicates a significant difference between the female WT and KRAS mice at the same age, p < 0.05. For different age compared to 5 weeks, the data were analyzed by one-way ANOVA (for normally distributed data) and Kruskal–Wallis H analysis (for non-normally distributed data). We adjusted the significance level α to 0.0125 according to the Bonferroni correction (0.05/4=0.0125). The symbols “A” and “a” indicate significant differences in the female WT and KRAS mice at 10, 15, 20 and 26 weeks relative to 5 weeks, p < 0.0125. [file peerj-08-10182-s004.png]

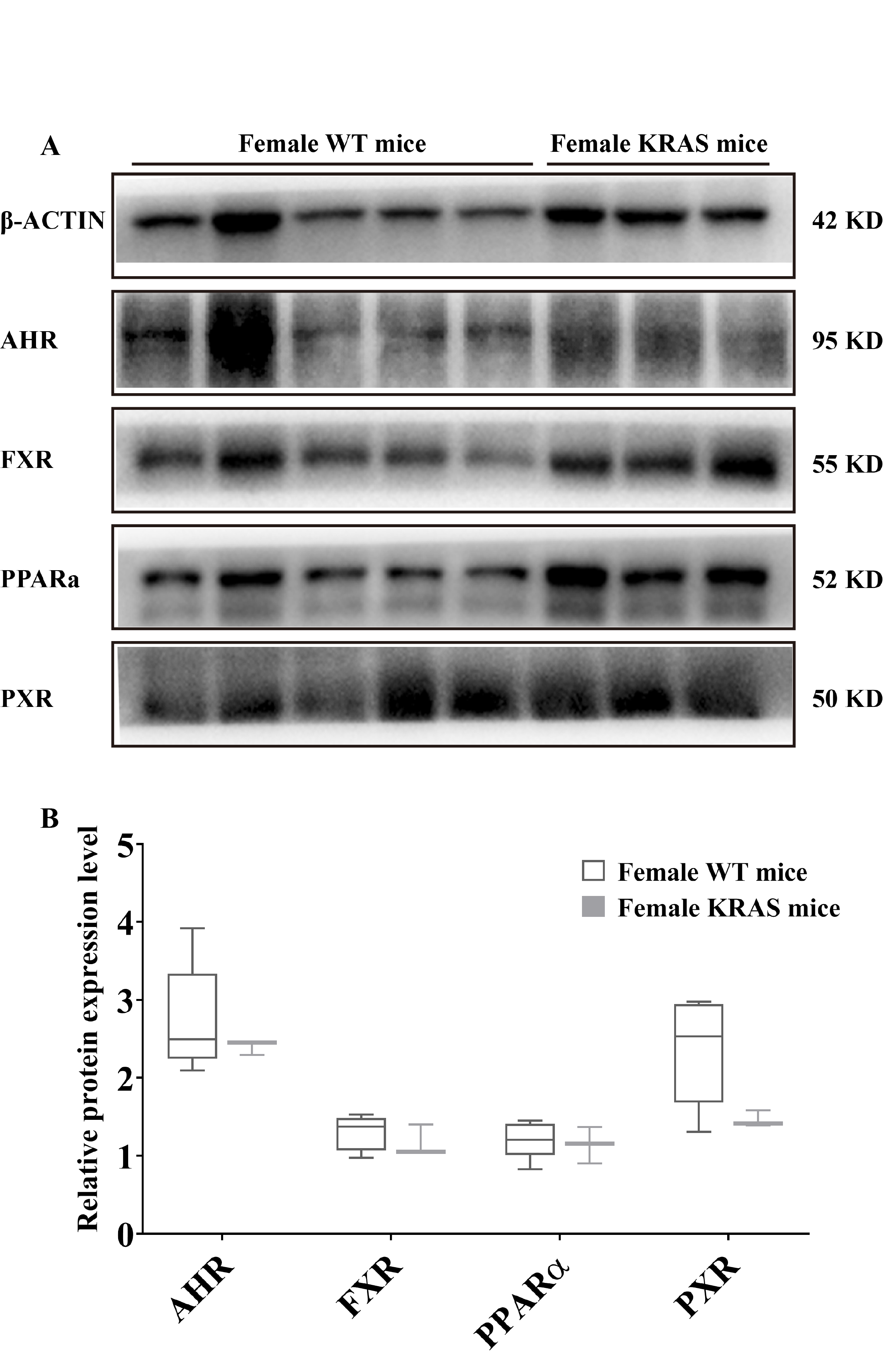

Supplement: Supplemental Information 5 — (A) The mprint of five proteins was represented and β-ACTIN was used as an internal control. (B) The data on protein expression levels was shown as a box chart. The data were analyzed by independent sample t tests (for normally distributed data) and Mann–Whitney U analysis (for non-normally distributed data). The symbol “*” indicates a significance difference of protein expression levels in the KRAS mice relative to that in the WT mice, p < 0.05. [file peerj-08-10182-s005.png]

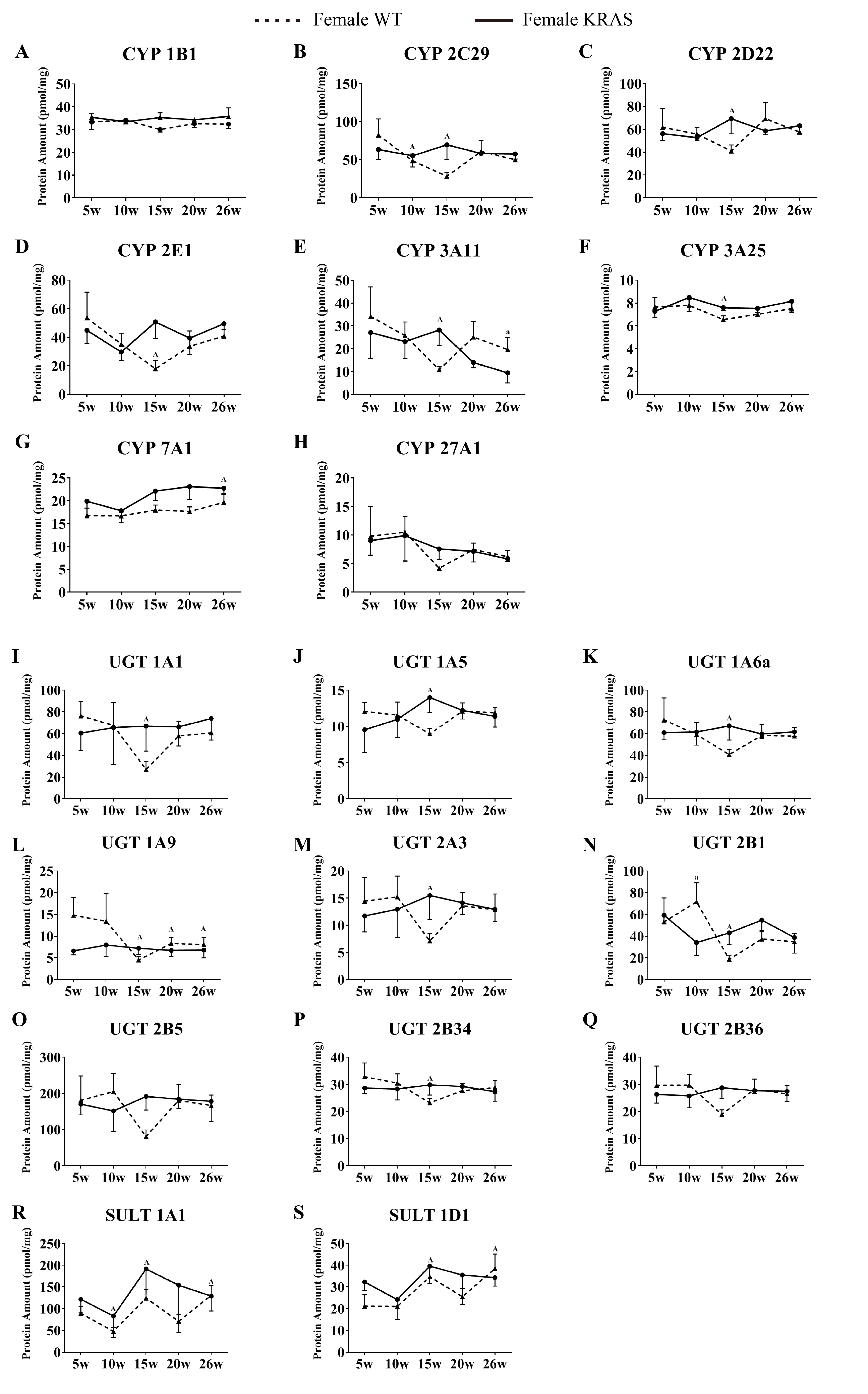

Supplement: Supplemental Information 6 — The dotted and solid lines represent the WT and KRAS mice, respectively. Each data point represents the mean ±SD. The data were analyzed by one-way ANOVA (for normally distributed data) and Kruskal-Wallis H analysis (for non-normally distributed data). We adjusted the significance level α to 0.0125 according to the Bonferroni correction (0.05/4=0.0125). The symbols “A” and “a” indicate significant differences in the male WT and KRAS mice at 10, 15, 20 and 26 weeks relative to 5 weeks, p < 0.0125. [file peerj-08-10182-s006.png]

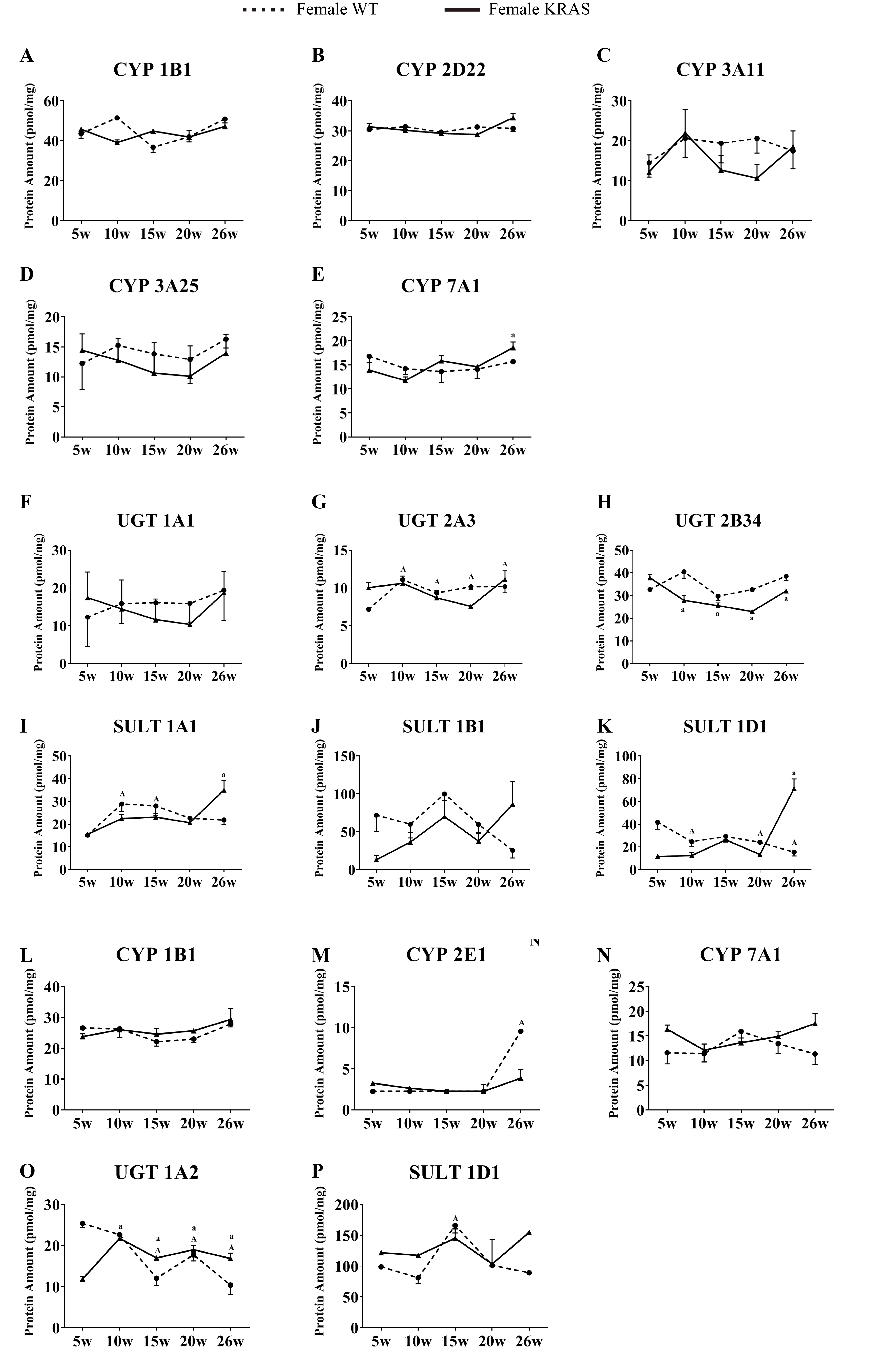

Supplement: Supplemental Information 7 — A-K shows the DMEs expression in intestinal tissue. L-O shows the DMEs expression in kidney tissue. The dotted and solid lines represent the WT and KRAS mice, respectively. Each data point represents the mean ±SD. The data were analyzed by one-way ANOVA (for normally distributed data) and Kruskal-Wallis H analysis (for non-normally distributed data). We adjusted the significance level α to 0.0125 according to the Bonferroni correction (0.05/4=0.0125). The symbols “A” and “a” indicate significant differences in the male WT and KRAS mice at 10, 15, 20 and 26 weeks relative to 5 weeks, p < 0.0125. [file peerj-08-10182-s007.png]

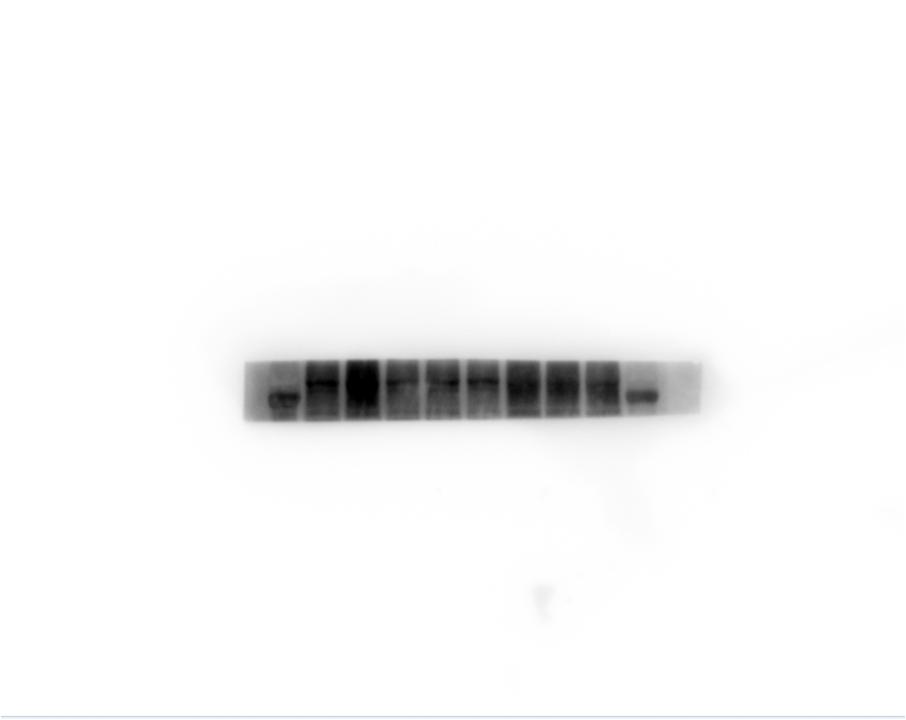

Supplement: Supplemental Information 9 [file peerj-08-10182-s009.zip › Raw data/Raw data-wb picture/female mice/AHR.png]

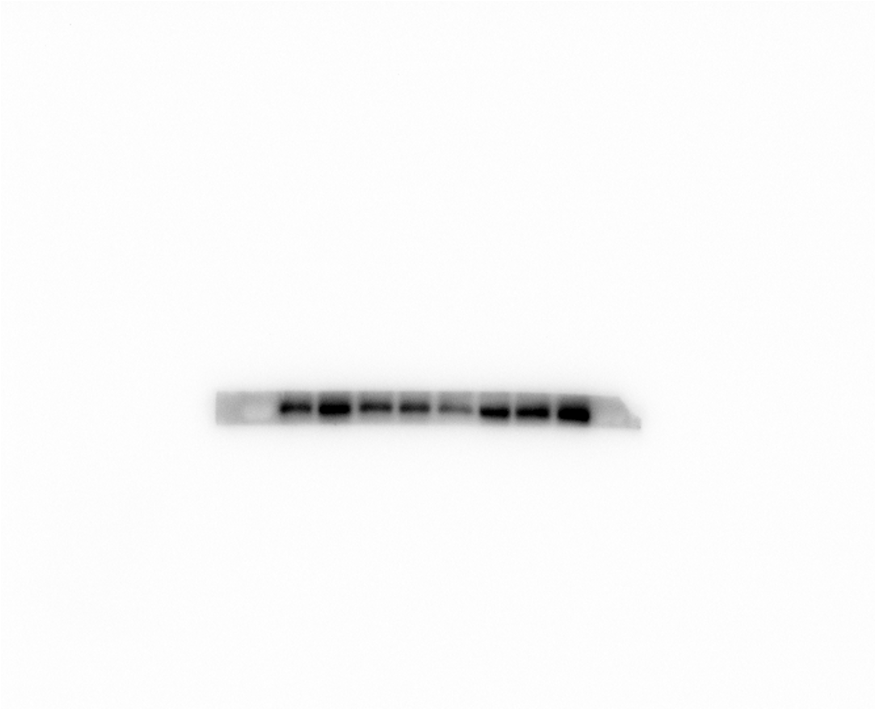

Supplement: Supplemental Information 9 [file peerj-08-10182-s009.zip › Raw data/Raw data-wb picture/female mice/FXR.png]

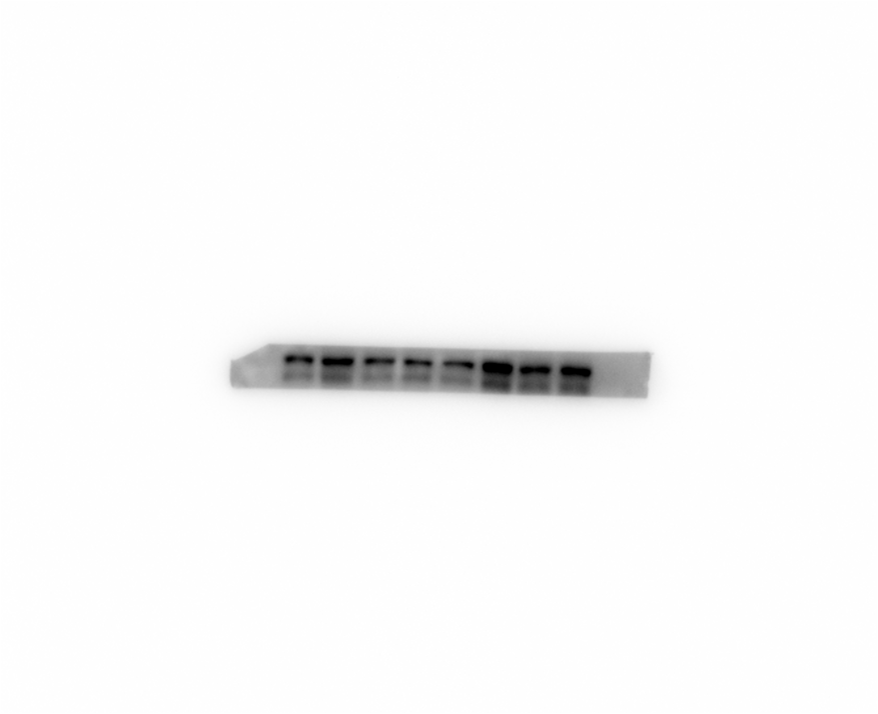

Supplement: Supplemental Information 9 [file peerj-08-10182-s009.zip › Raw data/Raw data-wb picture/female mice/PPARa.png]

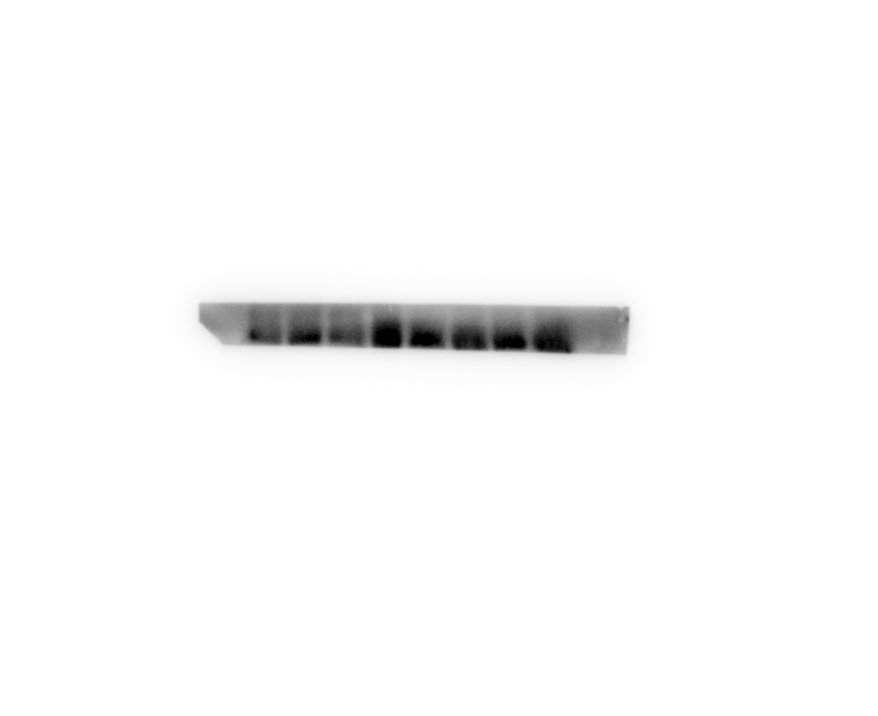

Supplement: Supplemental Information 9 [file peerj-08-10182-s009.zip › Raw data/Raw data-wb picture/female mice/PXR.png]

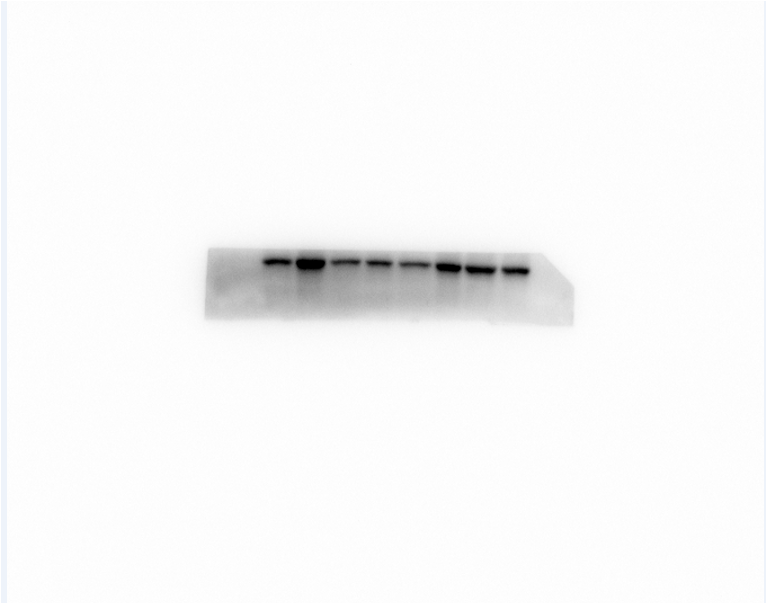

Supplement: Supplemental Information 9 [file peerj-08-10182-s009.zip › Raw data/Raw data-wb picture/female mice/a┬-ACTIN.png]

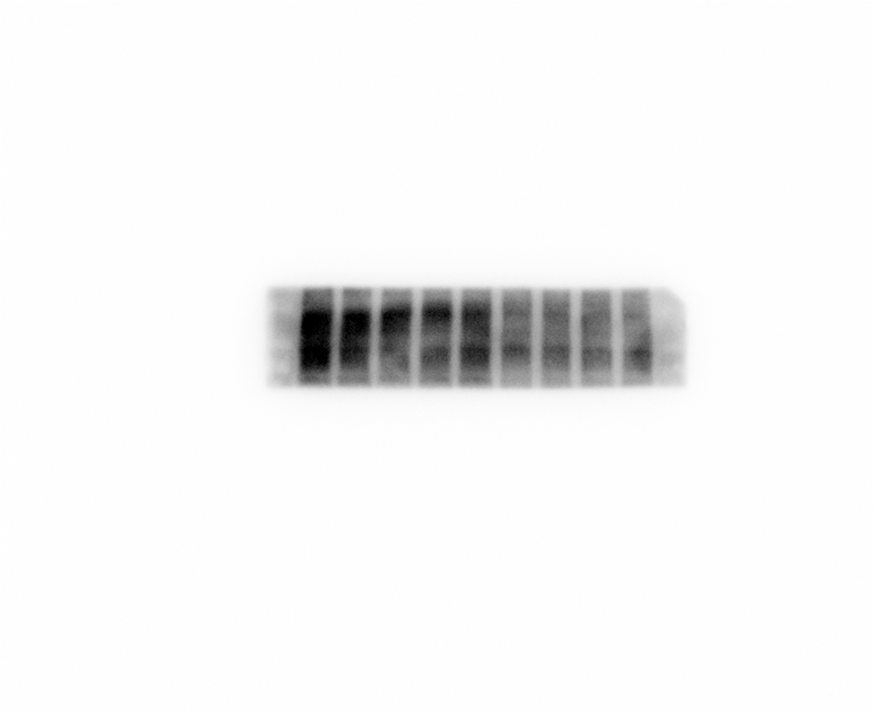

Supplement: Supplemental Information 9 [file peerj-08-10182-s009.zip › Raw data/Raw data-wb picture/male mice/AHR.png]

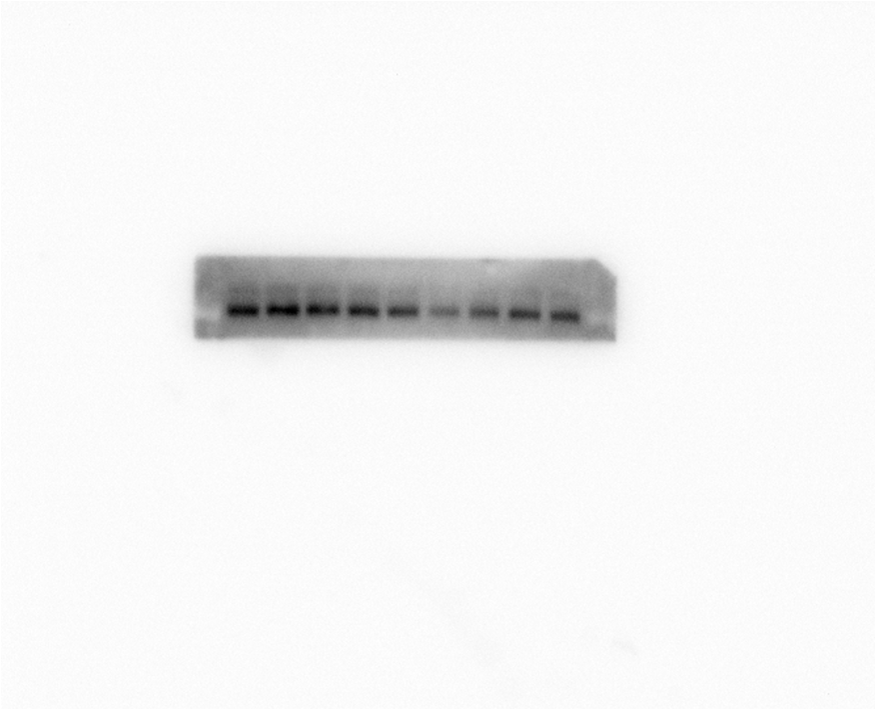

Supplement: Supplemental Information 9 [file peerj-08-10182-s009.zip › Raw data/Raw data-wb picture/male mice/FXR.png]

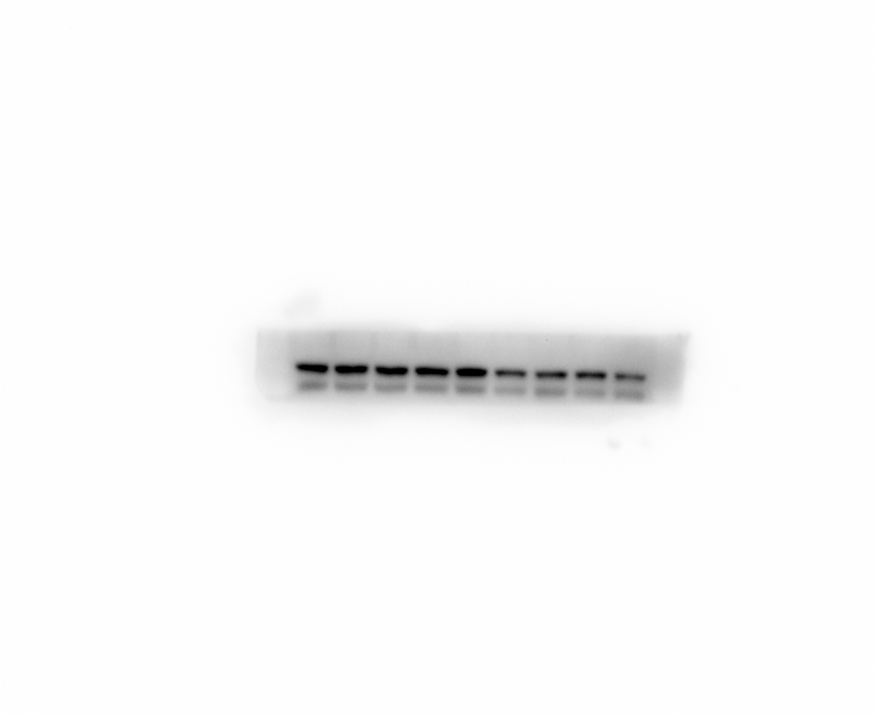

Supplement: Supplemental Information 9 [file peerj-08-10182-s009.zip › Raw data/Raw data-wb picture/male mice/PPARa.png]

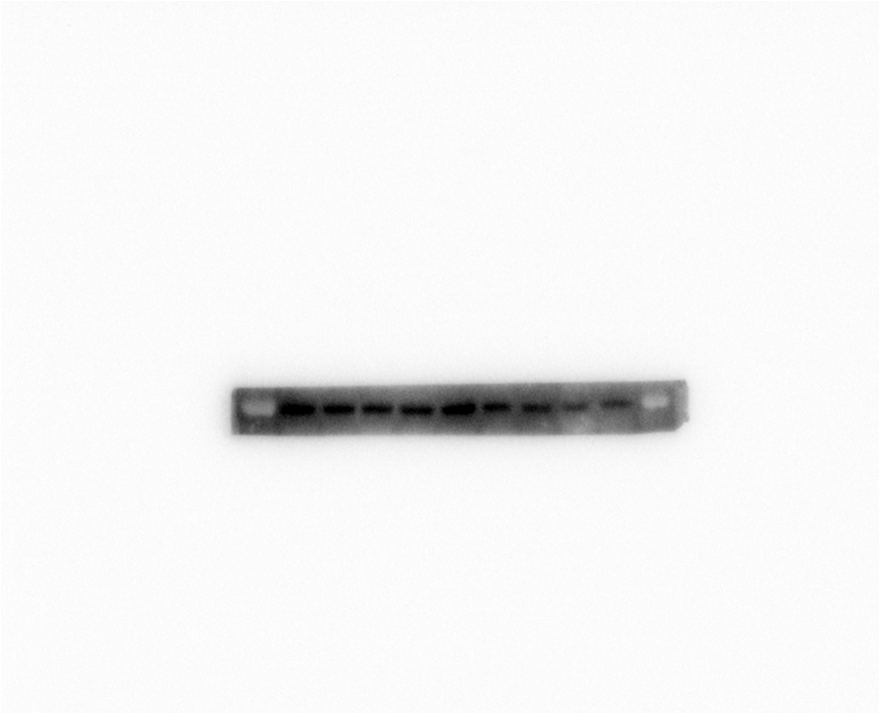

Supplement: Supplemental Information 9 [file peerj-08-10182-s009.zip › Raw data/Raw data-wb picture/male mice/PXR.png]

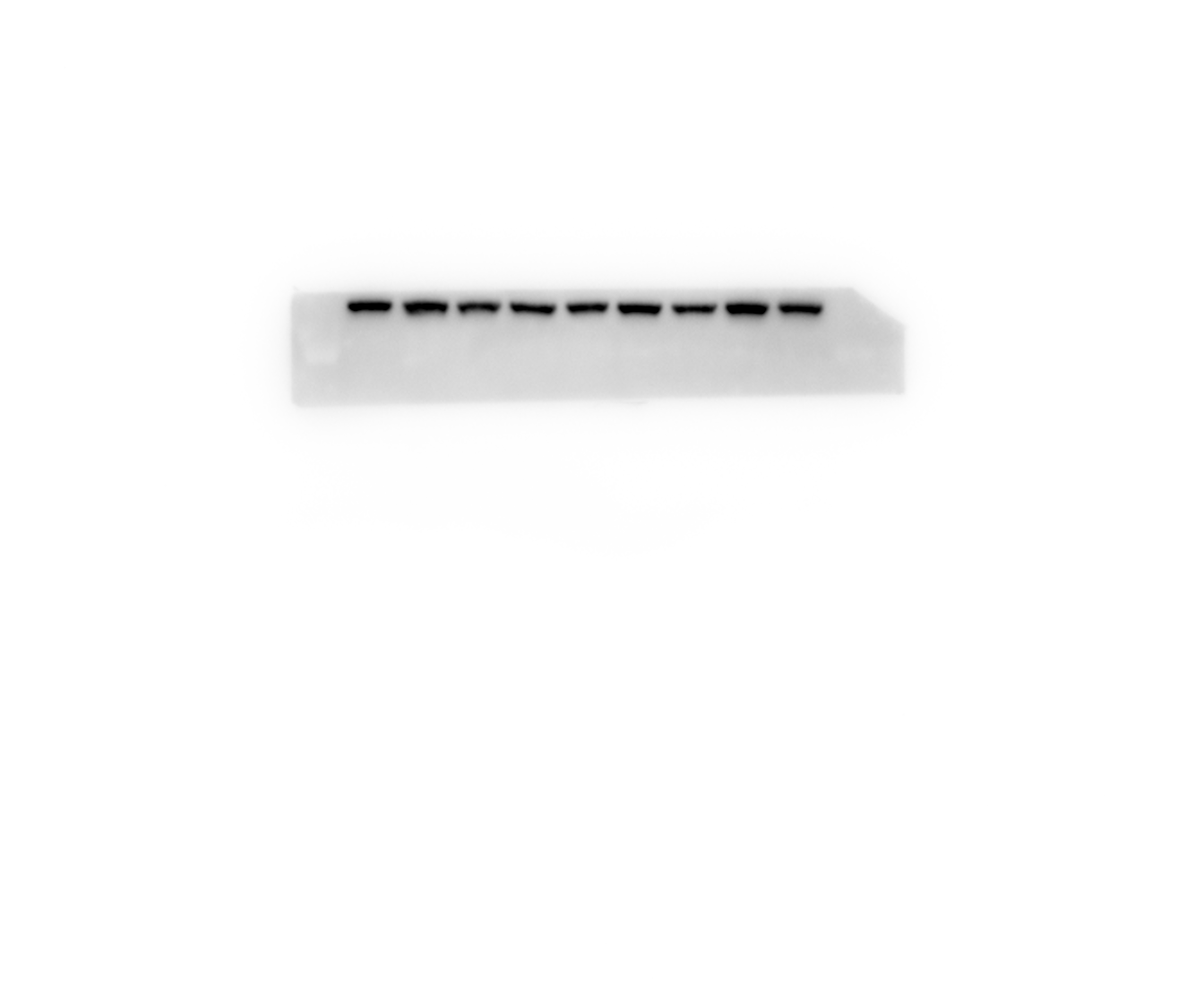

Supplement: Supplemental Information 9 [file peerj-08-10182-s009.zip › Raw data/Raw data-wb picture/male mice/a┬-ACTIN.png]
